# Poverty and a child’s height development during early childhood: A double disadvantage? A study of the 2006–2009 birth cohorts in Flanders

**DOI:** 10.1371/journal.pone.0209170

**Published:** 2019-01-02

**Authors:** Ronan Van Rossem, Isabelle Pannecoucke

**Affiliations:** 1 Department of Sociology, Universiteit Gent, Ghent, Belgium; 2 Flemish Housing Council, Brussels, Belgium; Centre Hospitalier Universitaire Vaudois, FRANCE

## Abstract

**Introduction:**

Poverty is a well-known risk factor for a child’s health and development. This paper aimed to establish whether poverty negatively affected both intra-uterine growth and early childhood growth, i.e., whether children facing poverty were at a double disadvantage.

**Methods:**

For this study, we made use of routinely collected data on child development throughout early childhood from the 2006–2009 birth cohorts in Kind & Gezin’s Ikaros database collected during 2,605,975 consultations with 273,935 children from birth to 730 days old. Indicators for child development at birth were gestational age and height-at-birth. A standardized height-for-age indicator captured height development throughout early childhood. A multidimensional indicator measured the risk of poverty. For the analysis of development at birth, we used linear and logistic regression; for the analysis of height development during early childhood, we estimated linear and logistic growth curve models.

**Results:**

The risk of poverty negatively affected both gestational age and height-at-birth. Throughout early childhood, we observed a negative relation between the risk of poverty and height-for-age indicators. However, the effect varied throughout childhood. Children at risk of poverty (over)compensated for their smaller stature at birth, and between ages 6 and 18 months, approximately, the negative effects of risk of poverty decreased substantially or disappeared. However, towards the end of the period studied, children born in households at risk of poverty started to lag again in height development.

**Conclusion:**

This study found that the risk of poverty indeed negatively affected a child’s growth, both in utero and in early childhood. However, the results suggest that developmental lags later in childhood were not merely an extension of such lags at birth.

## Introduction

Growth retardation in early childhood is indicative of health and developmental problems. It reflects the chronic exposure to risk factors and stressors, such as illness, poor nutrition, an unhealthy environment, etc. [1–4]. Developmental problems in (early) childhood are in turn predictive of health problems later in life as well as of psychological and cognitive problems and low educational and occupational achievement [5–12]. Once such developmental deficits occur, it may prove difficult to catch up and, because of continued exposure to risk factors, the health deficit may further increase with age. Early detection and treatment of developmental problems therefore is crucial.

This paper looks at the effects of poverty on the height development from birth to the age of 2 years in four Flemish birth cohorts (2006–2009). The main research question is whether children born in households facing poverty are at a double disadvantage regarding height development: 1) are they already at a disadvantage at birth compared to children born in non-poor households, and 2) do they experience an increased risk for further delays in height development during early childhood?

Already before birth, socioeconomic inequalities affect child health and development, including intra-uterine and post-natal growth [13–18]. Multiple pathways link poverty to height development. Although poverty affects both growth in utero and in early childhood, these are two distinct processes. The child’s intra-uterine growth is affected by the mother’s health and nutrition, her exposure to environmental risk factors (pollution, poor housing, etc.) and use of pre-natal and other medical care, as well as lifestyle choices (including substance use and smoking) [14, 18–20]. Living in poverty also generates a chronic (toxic) stress which affects the mother’s physical and psychological health [7, 21–23]. Pregnant women living in poverty tend to have higher exposure to such risk factors which lead to poorer birth outcomes, including being shorter at birth, but also premature birth, lower birth weight, etc. [14, 18, 20, 24, 25].

Low height-for-age in early childhood may be a consequence of a shorter height-at-birth. It takes time to overcome that initial disadvantage. Moreover, the chronic exposure during early childhood to risk factors that negatively affect height development remains higher for children born to low-income households than for those born in wealthier ones, which further reinforces socioeconomic inequalities in height-for-age [26–32]. Several studies confirm that in Belgium as well, children born in poor households on average have poorer health and lag in development compared to children from wealthier households [33–38].

A recent study examining the effects of socioeconomic status (SES) on height development during early childhood in the Netherlands, however, found that although lower-SES children tended to be smaller at birth than higher-SES ones, they tended to catch up quickly and often even overcompensated for their initial lag in development [39]. This suggests a discontinuity between the effects of SES and poverty on developmental gaps present at birth and developmental gaps acquired later in childhood.

## Methods

### Database

This study uses data from an administrative database, Ikaros, collected by Kind & Gezin. Given that this study makes use of anonymized data no ethical clearance was required. The use of the data was approved by the Flemish Oversight Committee for Electronic Administrative Data Traffic (http://vtc.corve.be/docs/beraadslagingen/VTC_beraadslaging_2014_04.pdf) and the federal (Belgian) Commission for the Protection of Privacy (https://www.privacycommission.be/sites/privacycommission/files/documents/beraadslaging_AG_024_2014.pdf). Kind & Gezin is a public agency in Flanders promoting the well-being of young children through a wide range of services. Registered nurses make regular home visits or provide in-office consultations to almost all families with newborn children residing in Flanders, and during these consultations anthropometric data is routinely collected. Although participation in the program is voluntary, almost all families with newborn children participate. Kind & Gezin estimates that in 2009 they reached 97.3% of their target population through home visits and 88.3% through in-office consultations [40]. At the initial visit, the staff apprises parents of the purpose of the consultations and informs them that participation is free of charge, is voluntary and can be terminated at any time. As Flemish law mandates both the consultation program and the data collection, and because Kind & Gezin health staff are bound by confidentiality rules, prior written informed consent was not collected from the participants. However, Kind & Gezin can share non-anonymized data with health professionals or hospitals only after explicit informed consent from the parents. Kind & Gezin carried out the anonymization of the dataset, which prevents the identification of siblings. This study used data of the 2006–2009 birth cohorts on *N*_*c*_ = 273,935 children and *N*_*o*_ = 2,837,285 observations, i.e., home visits or in-office consultations. The mean number of consultations per child was 10.36 (SD = 3.74), with a low of 1 consultation and a high of 45. The mean follow-up time was 649.33 days (SD = 312.61), with a maximum of 1,856 days. This study only considered observations of children up to age 730 days (2 years); 56.3% of the children were followed for up to 730 days or more. The effective sample size for the number of observations is *N*_*o*_ = 2,605,975. The mean number of consultations from birth to 730 days for each child was 9.52 (SD = 3.30).

### Variables

Anthropometric measures are used for the evaluation of physical development at birth and in early childhood. Two indicators of the child’s development at birth are included: gestational age and height-at-birth. Gestational age is measured in weeks. Dichotomous indicators were constructed for preterm births (< 37 weeks) and very preterm births (< 32 weeks). Height-at-birth was measured in centimeters. Two dichotomous indicators were created that capture being short-at-birth (< 46 cm) and very-short-at-birth (< 44 cm). All programming code and documentation is available at DOI: 10.17605/OSF.IO/RT4JA.

The child’s height development over its first 730 days is measured by the child’s height-for-age. This indicator was standardized for age and sex using the WHO LMS method [41–43]. However, as Flemish children follow a different developmental pattern than assumed by the WHO standards (see S1 Fig) we regressed the WHO values for height-for-age—separately for boys and girls—on age, using a fourth power polynomial, and new standardized scores were calculated as the deviation from this polynomial. Separate indicators were constructed for being short-for-age (height-for-age < –2SD), and for being tall-for-age (height-for-age > 2SD).

A main task of Kind & Gezin is to offer support to families with an increased risk of poverty [44]. Risk of poverty is not defined simply as material deprivation but as a limitation of the opportunities to fully participate in society [45]. Kind & Gezin developed a multidimensional risk-of-poverty indicator based on six criteria that are linked with an increased risk of poverty: 1) *household income*: a household with an irregular or disposable income lower than the poverty threshold; 2) *educational level of the parents*: one of the parents has achieved only an elementary-, vocational- or special education-level diploma, or did not complete secondary education; 3) *labor market participation of the parents*: both parents (or in the case of a single-parent household, a single parent) are in a precarious employment situation, are unemployed or employed in the subsidized social economy; 4) *parental difficulties in caring for the child and/or insufficient stimulation of the child*; 5) *quality of the housing*: a poor quality, unhealthy or unsafe dwelling lacking basic amenities; and 6) *health of the household members*: one or more household members is in poor health, has a chronic disease or is disabled, or one underutilizes health services.

During the initial visit, the nurses checked which criteria are met. They do not use a strict checklist, but are trained to recognize indicators for the various dimensions and base their decisions on observations and on interviews with the parents. Kind & Gezin [46] considers a child at risk of poverty when at least three of the criteria are met. For this study, we created a *risk-of-poverty* indicator by counting the number of criteria met. We distinguish four levels of risk of poverty: no risk (0 criteria), low risk (1 criterion), medium risk (2 criteria) and high risk (3+ criteria). As the Kind & Gezin definition of risk of poverty only requires three out of six criteria to be met, Kind & Gezin staff often stopped recording criteria once three were identified; we cannot therefore reliably distinguish between poverty levels when more than three criteria have been met.

The following confounding variables are included in the analysis: the sex of the child (0: boy, 1: girl), the age of the mother (in years) at birth of the child, the birth order of the child and the region where the mother was born, with as categories, identifying the main migrant groups and the geographic distribution of the migrants: 1) Belgium, 2) Turkey, 3) Morocco, 4) Northern and Western Europe, Northern America, Australia and New Zealand, 5) Southern Europe, 6) Eastern Europe, 7) South and Central America, 8) Asia and Oceania, and 9) Africa. Note that this indicator only partly captures the mother’s ancestry, as all second or higher generation migrant mothers are Belgian born. Gestational age is included as a control variable in the analysis for the height-development indicators. The age of the child (in days) is included in the analyses for height development during early childhood (≤ 730 days).

### Statistical analysis

To estimate the effects of risk of poverty on birth characteristics, ordinary least squares linear regression was run for the metric dependent variables (gestational age and height-at-birth), and logistic regression for the dichotomous dependent variables (preterm, very preterm, short-at-birth, very-short-at-birth), with risk of poverty as the main independent variable (reference: no risk, 0 criteria) and age of mother (linear and squared), birth order and region of mother’s birth as control variables. For the height-at-birth variable, a second model was run as well, including gestational age as a control variable. Adjusted means and proportions by risk of poverty were calculated for all analyses. These are the point estimates for fictitious observations that differ on the risk-of-poverty indicator and score the mean on all other variables in the model.

To track the development of the children by risk of poverty throughout early childhood, growth curves were estimated using linear models for height-for-age and logistic models for short-for-age and tall-for-age. Three consecutive models were run for each of the outcomes. The first model estimated the zero-order effect of risk of poverty on child development. For the dichotomous outcomes, this model also included sex and age. As height-for-age had already been adjusted for age and sex, there was no need to control for these variables. The dichotomous variables, in contrast, capture extreme scores and thus the spread of the observations that do not need to be independent of age and sex. In the second model, the other control variables—age of mother, region of mother’s birth, birth order and gestational age—were added. These first two models are main effect models and assume that the effect of risk of poverty remains stable over the entire period (730 days) studied. In the third model, the effect of risk of poverty varies according to age and includes an interaction between risk of poverty and age. Given the small proportion of missing data, all analyses were complete cases only.

## Results

The sample contained more boys than girls (sex ratio = 1.05, see Table 1). No significant sex differences were observed (at *p* < 0.010) for either risk of poverty or the control variables. Most of the children (83.5%) were born in households with no real risk of poverty (0 criteria), and only 6.2% met the Kind & Gezin criteria for (high) risk of poverty (3+ criteria). The average age of the mother at the birth of the child was 29.2 years, while only 1.0% of the children were born to mothers 18 years old or younger, and 1.2% to mothers over 40 years old. Firstborns accounted for 47.2% of the children; only 2.1% were fifth or higher in birth order. The highest birth order observed in the sample was 18.

**Table 1:** Descriptive statistics for risk of poverty and child birth characteristics; control variables measured at the children’s level.

	Boys	Girls	Total	*p*_diff_	*N*
Risk of poverty				0.209	273,619
0	83.46%	83.51%	83.48%		
1	7.72%	7.69%	7.71%		
2	2.69%	2.58%	2.64%		
3+	6.13%	6.22%	6.17%		
Age of mother ^a^	29.19 (4.83)	29.21 (4.82)	29.20 (4.83)	0.168	272,013
Birth order ^a^	1.80 (1.02)	1.81 (1.03)	1.81 (1.03)	0.253	273,487
Region of mother's birth				0.033	267,634
Belgium	79.34%	79.04%	79.20%		
Turkey	2.88%	2.93%	2.90%		
Morocco	4.71%	4.92%	4.81%		
Northern Europe + Western Europe + Northern America + Australia + New Zealand	2.94%	2.95%	2.95%		
Southern Europe	2.00%	2.09%	2.04%		
Eastern Europe	2.28%	2.31%	2.29%		
South and Central America	0.68%	0.64%	0.66%		
Asia + Oceania	2.57%	2.46%	2.52%		
Africa	2.60%	2.65%	2.63%		
Gestational age ^a^	38.86 (1.90)	38.94 (1.86)	38.90 (1.88)	0.000	273,042
Preterm < 37 wks	7.74%	7.03%	7.39%	0.000	273,042
Very preterm < 32 wks	0.99%	0.93%	0.96%	0.087	273,042
Height-at-birth ^a^	50.19 (2.62)	49.40 (2.55)	49.80 (2.62)	0.000	269,454
Short < 46 cm	4.24%	5.90%	5.05%	0.000	269,454
Very short < 44 cm	1.83%	2.22%	2.02%	0.000	269,454
*N*	140,109	133,510	273,619		
%	51.21%	48.79%	100.00%		

^a^ For metric variables, mean and (standard deviation) are provided.

*p*_*diff*_: refers to significance level of a *t*-test for metric variables and a *χ*^2^-test for categorical variables.

Although there were no significant sex differences on the background characteristics of the children, we observe significant differences by sex for birth characteristics. Boys overall had a somewhat shorter gestational time than girls which also translated into a somewhat higher risk for preterm birth with an odds ratio (OR) of 1.11. The OR compares the risk of an outcome for a given risk of poverty to that of children at no risk of poverty. An OR of 1 means the risk for the outcome in the group with a given risk of poverty does not differ from that in the group with no risk of poverty. An OR greater than 1 means the risk for the outcome is higher than in the group at no risk of poverty, and an OR less than 1 that the risk for the outcome is lower than in the group at no risk of poverty. Boys, however, tended to be somewhat taller at birth than girls. This implies that, compared to boys, girls were at an increased risk of being short-at-birth (OR = 1.42) or very-short-at-birth (OR = 1.22).

### Poverty and birth characteristics

**Fig 1:**
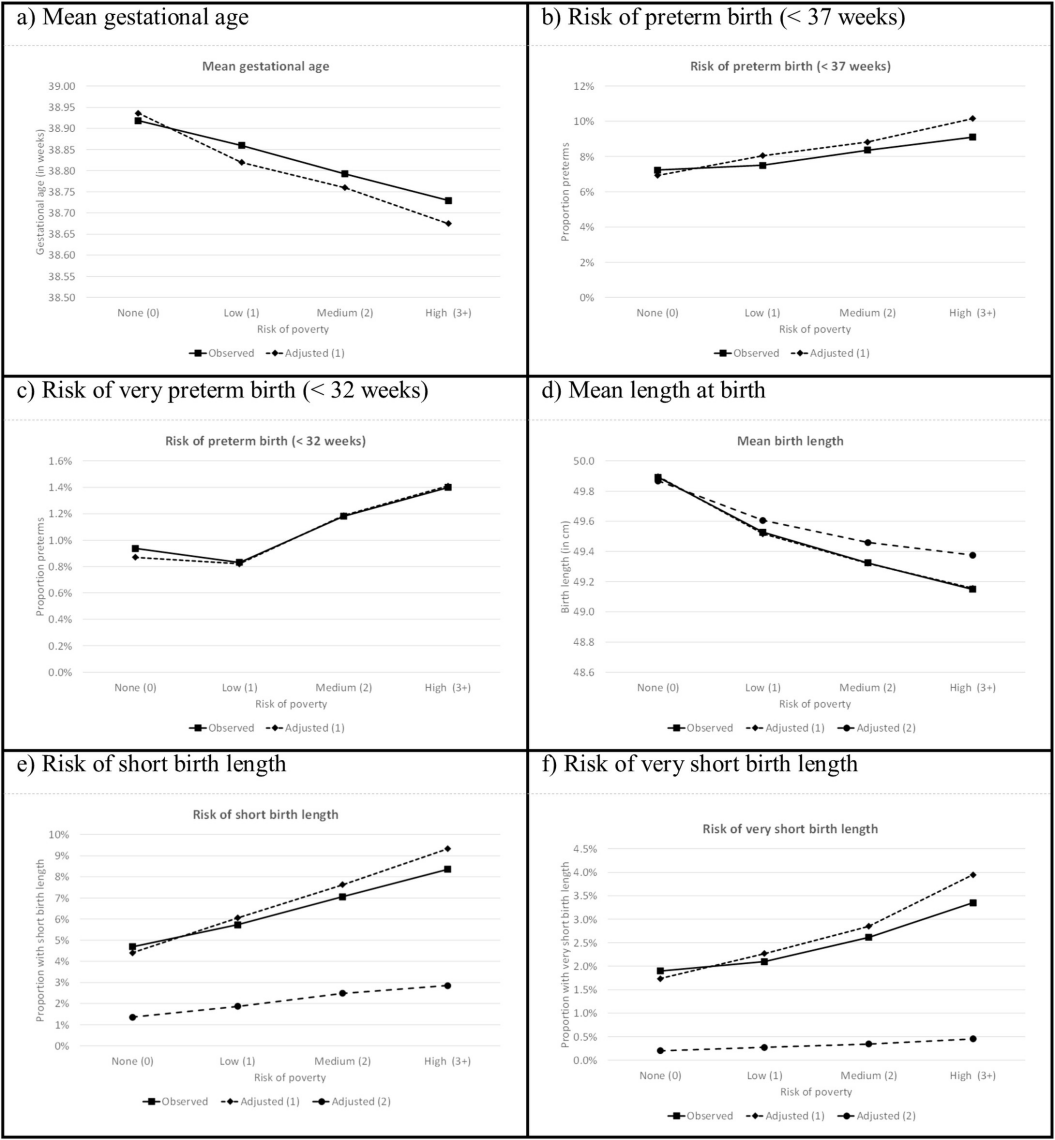
Linear and logistic regression estimates of effects of risk of poverty on gestational age, preterm birth, very preterm birth, mean height-at-birth, short-at-birth and very-short-at-birth, observed and adjusted. (1) Adjusted for sex of child, birth order, age of mother and region of mother’s birth. (2) Adjusted for sex of child, birth order, age of mother, region of mother’s birth and gestational age. Estimation of the effects of risk of poverty on gestational age and mean height-at-birth based on linear regression, and on risk of preterm birth, very preterm birth, short birth height and very short birth height based on logistic regression.

The risk of poverty had a clear effect on the gestation period. Children born in households with a high risk of poverty (3+ criteria) had on average a gestation period 32 hours shorter than those born in households at no risk of poverty (0 criteria) (see Fig 1A). The risk of preterm or very preterm birth increases with the risk of poverty: 7.2% of children with no risk of poverty were preterm compared to 9.1% of children at high risk for poverty; their risks of very preterm birth were 0.9% and 1.4%, respectively (both differences: *p* < 0.001). When controlling for the sex of the child, its birth order, the mother’s age and region of birth, the effects of risk of poverty on gestational age and on the probability of preterm birth increased further (see S1 and S2 Tables). For instance, the difference in estimated mean gestational age between children at high risk of poverty and those at no risk increased to 44 hours, while the OR for preterm birth increased from 1.29 to 1.52 and for very preterm birth from 1.50 to 1.63.

The risk of poverty had a negative effect on the average height of the child. Children born to households with a high risk of poverty were on average 7 mm shorter at birth than those born in a household with no risk of poverty (see Fig 1D and S2 Table). Controlling for the sex, birth order and the mother’s age and region of birth did not alter this finding. Only when the gestational age of the child was added as a control variable was the average difference between children born in a household with a high risk of poverty and those with no risk of poverty reduced to 5 mm.

Likewise, children from households with a risk of poverty were more likely to be short (< 46 cm) or very short (< 44 cm) compared to children not at risk of poverty. For children at high risk of poverty the ORs for being short or very short compared to children not at risk of poverty were 1.85 (*p* < 0.001) and 1.79 (*p* < 0.001), respectively. However, when controlling for the child’s sex and birth order and the mother’s age and region of birth, the estimated differences in risk of being short-at-birth or very-short-at-birth between children at no risk of poverty and those at some risk of poverty increased. While the estimated risk for children at no risk of poverty declined, it increased in the three other categories (see S1 and S2 Tables). Again, gestational age proved an important predictor of being short-at-birth or very-short-at-birth, but even after controlling for it, the effect of risk of poverty on these outcomes remained significant, and the higher the risk of poverty, the higher the chance of being short-at-birth or very-short-at-birth.

### Height development in early childhood

Overall, the height-for-age measurements proved consistent over time, with an intra-class correlation coefficient of 0.69 (*p* < 0.001). The observed stability confirms that because of prematurity and smaller stature at birth, children at risk of poverty begin life already handicapped: shorter height-at-birth increased children’s risk for a low height-for-age over subsequent observations.

**Fig 2:**
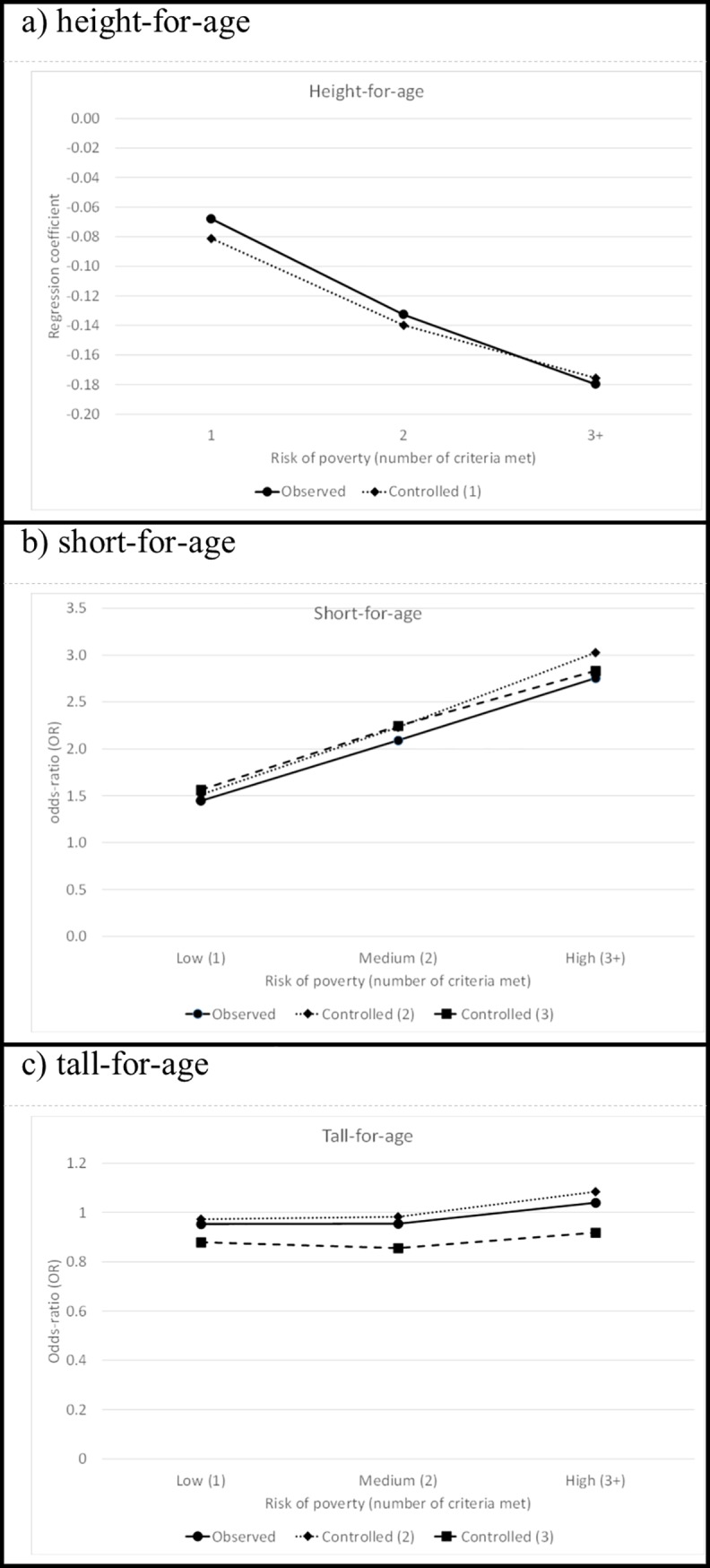
Linear and logistic growth curve estimates for the effects of risk of poverty on height-for-age, short-for-age and tall-for-age during early childhood (age ≤ 730 days), main effects only. (1) Adjusted for gestational age, birth order, age of mother and region of mother’s birth. (2) Adjusted for sex of child and age of child. (3) Adjusted for sex of child, age of child, birth order, age of mother, region of mother’s birth and gestational age. Adjusted effects for height-for-age are based on linear growth curve models, and are based on logistic growth curve analysis for short-for-age and tall-for-age.

The analyses confirmed that children born in households at risk of poverty (low, medium or high) during their first two years were on average smaller than children born in households with no risk of poverty (see Fig 2A). The effect on height-for age increased with the risk of poverty and remained robust when controlling for gestational age, birth order, mother’s age and region of birth (see S3 and S4 Tables). Children at low risk of poverty scored on average 0.081 lower on height-for-age than children not at risk of poverty, 0.140 lower than children with a medium risk of poverty and 0.176 lower than children with a high risk of poverty (all *p* < 0.001). Likewise, the results show that with an increase in the risk of poverty, the risk of being short-for-age increased.

**Fig 3:**
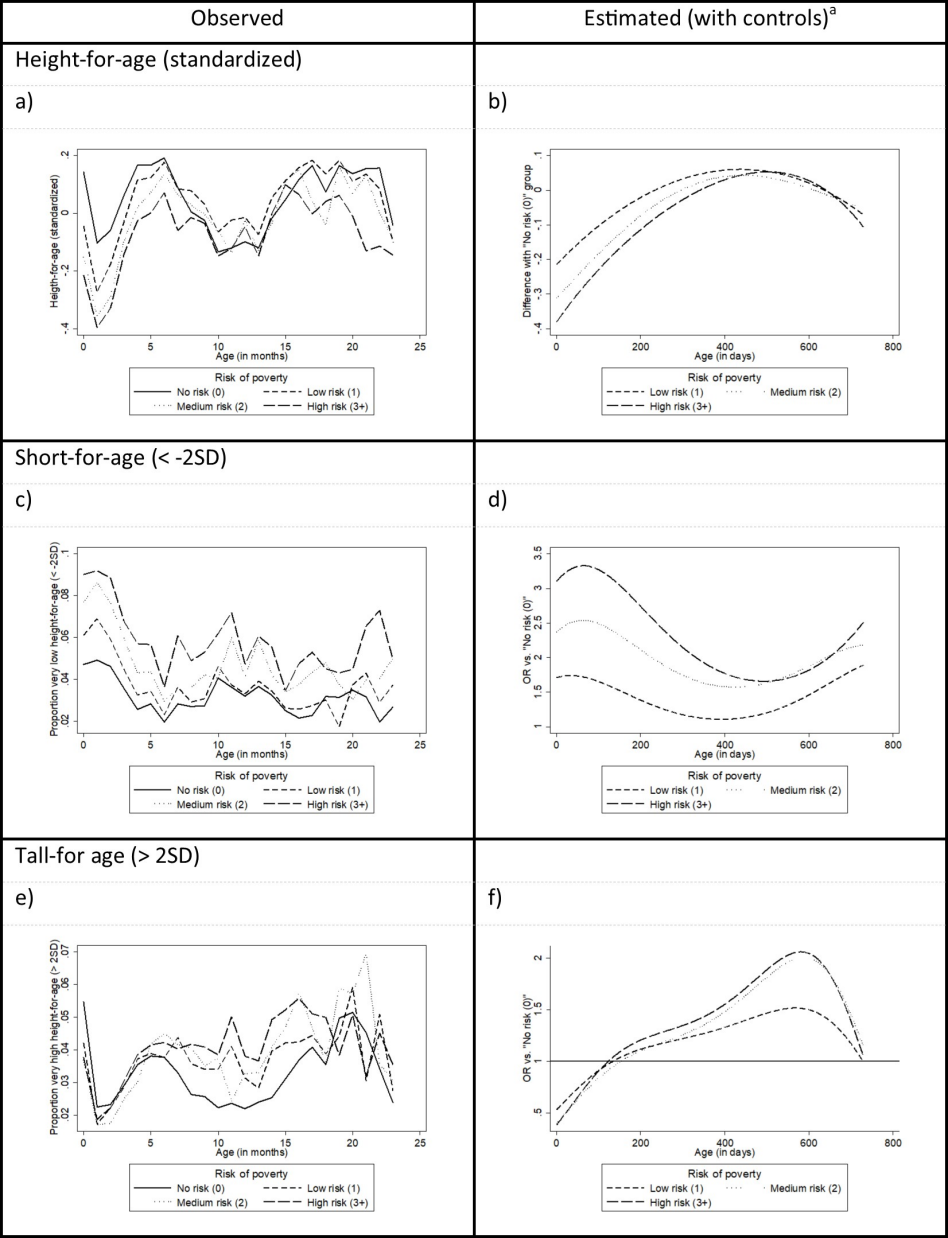
Linear and logistic growth curve estimates of the age-dependent effects of risk of poverty on height-for-age, short-for-age and tall-for-age. ^a^ Adjusted for sex of child, birth order, age of mother, region of mother’s birth and gestational age. Age is included as a fourth degree polynomial. Estimated results based on linear growth curve models for height-for-age and on logistic growth curve models for short-for-age and tall-for-age.

Fig 3A shows the monthly average standardized height-for-age by risk of poverty and illustrates that the risk-of-poverty effect on height-for-age varied throughout early childhood. Although, on average, children with some risk of poverty both at birth and during the first few months of life were smaller than children at no risk of poverty were, they soon caught up. We no longer observed a significant difference (at *p* = 0.050) in height-for-age between children with a low or medium risk of poverty and the children at no risk of poverty in month 6, while children at high risk of poverty caught up during month 7. Between months 8 and 15, children with a low risk of poverty were even significantly taller for their age than children at no risk. Children at a high risk of poverty, in contrast, lagged in height again from month 17 on compared to children at no risk of poverty. An estimation controlling for confounding variables (see Fig 3B and S5 Table) of height-for-age difference by risk of poverty during early childhood also shows that height differences gradually decreased, and that from about 8 months on, children with a low risk of poverty were generally even somewhat taller than children at no risk of poverty. Children at medium risk of poverty caught up around month 10 and those at high risk of poverty at approximately a year. At about 20 to 21 months of age, children with any risk of poverty were expected to start lagging again in height development compared to children at no risk of poverty.

### Short-for-age

The risk of poverty also increased the risk of being short-for-age during early childhood; this effect became stronger with a higher risk of poverty (Fig 2B and S4 Table). Although the observed OR of being short-for-age for children with a low risk of poverty compared to children at no risk of poverty was 1.45, it increased to 2.09 for children at a medium risk for poverty and to 2.75 for children at high risk of poverty (all *p* < 0.001). Controlling for other child and mother characteristics did not substantially alter the effects of risk of poverty (see S3 and S4 Tables).

The observed risk for being short-for-age by risk-of-poverty also varied over early childhood (Fig 3C). In the first months of life, children with any risk of poverty were at an increased risk for being short-for-age, but by month 5, this increased risk had already disappeared for children with a low risk of poverty. The difference between children at no risk of poverty and children at a medium risk of poverty became non-significant in month 7, but up to month 17, sometimes significant differences in risk for being short-for-age were observed. For those at high risk of poverty, catching up occurred only at month 18 and was quite short-lived: from month 21 on, they were again already at a significantly higher risk of being short-for-age than children at no risk of poverty were. For children with a low or medium risk of poverty, a similar but less pronounced trend was observed. However, the difference in risk between these groups and those at no risk became significant only during the final month (23) observed. The estimated results (Fig 3D and S5 Table), controlling for the sex of the child, gestational age and the mother’s age and birth region, show a similar picture.

### Tall-for-age

On average, we found no differences in the risk of being tall-for-age based on the risk of poverty (Fig 2C). Only when we controlled for the sex and age of the child did the risk of being tall-for-age for children at high risk of poverty compared to those at no risk become marginally significant but positive (OR = 1.08, *p* < 0.050). When additional controls were added, the effects of risk of poverty on the risk of being tall-for-age became negative; this was almost exclusively due to the mother’s region of birth (see S3 and S4 Tables). The estimated OR for being tall-for-age for children at low risk of poverty compared to children at no risk was OR = 0.88 (*p* < 0.001); for children at medium and high risk it was, respectively, OR = 0.85 (*p* < 0.001) and OR = 0.92 (*p* < 0.050).

However, when the results were broken up by the age of the child, they revealed a much more complex picture. Although children at risk of poverty at birth indeed tended to have a lower risk of being tall-for-age than children at no risk of poverty did, they caught up during the first few months (Fig 3E). From month 7 through month 16, children at risk of poverty even had a higher risk for being tall-for-age than children at no risk of poverty did. After 16 months of age, the differences based on risk of poverty again disappeared. The estimated model (Fig 3F and S5 Table) shows a similar trend. At about month 6, children at any risk of poverty caught up with children at no risk of poverty concerning the probability of being tall for their age; from 6 months to 2 years, their OR of being tall-for-age compared to children at no risk of poverty remained greater than 1. By age 2, however, these differences again disappeared.

## Conclusion and discussion

### Aim and main findings

This paper examined the effects of being born in a household at risk of poverty on a child’s height development during early childhood (up to 730 days of age). The underlying hypotheses were that already at birth, children at risk of poverty will lag those not at risk in height development, and that exposure to the risk of poverty during early childhood will further increase this gap. These effects were expected to increase in magnitude as the severity of the risk of poverty increased.

The results confirm that being born in a household at risk of poverty on average leads to a lower height-at-birth and increases the risk of being short-at-birth. These effects increase with the risk of poverty. However, the analyses also show that much of the observed differences in height-at-birth or risk of being short-at-birth based on risk of poverty can be explained by differences in gestational age. With the risk of poverty, average gestational age decreases and the risk for prematurity increases. However, even after controlling for this and other risk factors, the risk of poverty negatively affects height-at-birth.

Given that children in households at risk of poverty tend to lag behind those not at risk regarding height-at-birth and tend to grow up under less favorable circumstances, we can expect these children to also be smaller during early childhood. The results confirm that the risk of poverty overall is negatively related with height-for-age during early childhood and with an increasing risk of being short-for-age and a decreasing risk of being tall. However, the effect of the risk of poverty on the height development of children varies over early childhood. Although children at risk of poverty do start at a disadvantage, they compensate quite rapidly, i.e., within the first six months of life, and catch up with children not at risk of poverty and may even grow taller. However, by the end of the period studied, children at risk of poverty again begin to fall behind those at no risk of poverty in height development. The reason behind this (over)compensation remains unclear, but one hypothesis is that poorer parents are less likely to breastfeed or may quit breastfeeding sooner [47, 48], which may lead to faster growth of the child [49, 50]. Bottle-feeding may provide more calorie-rich nutrition but is not necessarily more balanced or healthier. By the end of the period studied, the negative effects of exposure to poverty-related environmental factors, including parental behavior, might again become apparent.

These findings suggest that the developmental lag at birth and in early childhood of children from poorer households is not continuous. The initial lag is probably due to the mother’s exposure to adverse conditions related to poverty, including nutritional factors, poor living (and working) conditions, poor health behaviors, less access to health care and poverty-related stress, all of which negatively affect her health and overall physical condition and thus, indirectly, affect the development of the fetus. Later in early childhood, poverty-related risk factors begin to directly affect the child, its health and its development.

### Limitations

When interpreting the data we have to take into account limitations related to the operationalization of risk of poverty to the data set used. Firstly, the main independent variable is the risk of poverty rather than poverty itself. Not all households defined as at risk of poverty actually experience poverty; therefore not all children defined as at risk of poverty will be exposed to its potentially adverse effects. Consequently, using a risk-of-poverty indicator results in underestimating the effects of poverty, and this underestimation increases with the proportion of false positives. The proportion of false negatives is considerably smaller.

We also operationalized the risk of poverty as a multidimensional concept. The indicator used captures the risk of poverty by counting the number of dimensions experienced by the household. The implied assumption in the analysis is that all dimensions (low income, low parental education, poor housing conditions, etc.) are interchangeable regarding their effect on a child’s growth.

Secondly, the Ikaros dataset is an administrative dataset to track children for consultancy services and thus contains no information on the mechanisms that may explain how poverty affects the growth development of children during early childhood or on other confounding factors, such as the height of the parents. The latter may be considered a proxy for the genetic component of height development but can also be interpreted as a proxy for the intergenerational transmission of poverty and its adverse health effects. The data is also collected by health workers for administrative purposes rather than scientific ones. Although trained to score the dimensions consistently, these health workers depend on their perceptions and evaluations rather than on hard data. This may introduce measurement error and bias in the risk-of-poverty indicators.

As participation in the Kind & Gezin program is voluntary, some self-selection may occur. However, as over 97% of eligible children do participate, any selection bias will be minor. Although parents may have many reasons for the non-participation of their children, it is certainly possible that children with severe developmental or health problems are overrepresented among the non-participants as these children are more likely to be followed up with by specialized health professionals. Similarly, the Ikaros data provides little insight into those who drop out. Families decide when to stop attending Kind & Gezin consultations. This may introduce a further self-selection bias. However, we found that children at risk of poverty were followed significantly (at *p* < 0.001) longer by Kind & Gezin and had more consultations. One possible explanation is that poorer households visit general practitioners or pediatricians less frequently for regular follow-ups regarding their child’s development. We also observe a small positive but significant correlation (*r* = 0.035, *p* < 0.001) between height-for-age and age at the last consultation. Children with obvious developmental delays or health problems are likely to be seen by other health professionals. This potential bias, although small, tends to be conservative, and preferentially removes children with developmental problems from the program.

### Generalizability

The generalizability of these findings remains limited to high-income countries where malnutrition is rare, even among households living in poverty, and where all families have, at least in theory, access to high-quality health care. In low-income countries, access to such nutrition and health services may be problematic, particularly for the lower socioeconomic strata. Here, catching up may not occur.

Even in a high-income region with a well-developed, comprehensive welfare state with quasi-universal access to good health care services, where malnutrition during early childhood is not a major problem, children born in a household at risk of poverty are still at an increased risk for slower height development, both at the intra-uterine stage and during the first two years of life. This study confirms the existence of a socioeconomic gradient regarding health inequalities that manifests at birth and continues throughout early childhood. Further research to discover and document the mechanisms through which household poverty affects child development in high-income regions, and which is beyond the scope of this project, is needed. This paper does show that children born to families at risk of poverty are indeed at a double disadvantage. These children begin life at a disadvantage, as they tend to have a greater risk of being shorter at birth, due in part to shorter gestation times. And although many children seem to overcome this initial lag in height development during their first year of life, later in early childhood they are exposed to new risk factors that slow their height development and cause them to again fall behind children born to households at no risk of poverty.

## Supporting information

S1 Fig Age and sex adjustment of standardized height-for-age scores: mean scores before adjustment and predicted means, and mean scores after adjustment. (PDF)

S1 Table Linear regression results for gestational age and height-at-birth, and logistic regression results for preterm birth, very preterm birth, short-at-birth, and very-short-at-birth. Significance: *: *p* < .050, **: *p* < 0.010, ***: *p* < 0.0001.
Models 1, 4a and 4b estimated using linear regression; models 2, 3, 5a, 5b, 6a and 6b estimated using logistic regression.
(PDF)

S2 Table Observed and adjusted means and proportions for birth characteristics by risk of poverty. (1) Adjusted for sex of child, birth order, age of mother and region of mother’s birth.
(2) Adjusted for sex of child, birth order, age of mother, region of mother’s birth and gestational age. Effects of risk of poverty are significant at p < 0.001 for all models.
Adjusted means were calculated for gestational age and height-at-birth; adjusted proportions for preterm < 37 weeks, preterm < 32 weeks, short-at-birth < 46cm, very-short-at-birth < 44 cm.
(PDF)

S3 TableRandom effects linear regression growth curve results for height-for-age, and random effects logistic regression growth curve results for short-for-age and tall-for-age.Significance: *: *p* < 0.050, **: *p* < 0.010, ***: *p* < 0.001.Values in cells are estimated linear or logistic regression coefficients and, between parentheses, the standard errors of the coefficients.$\ln\left(\sigma_{\nu }^{2}\right)$: child level variance component, component value and, between parentheses, its standard error.*ρ*: proportion total variance due to child level variance component.(PDF)

S4 Table Observed and adjusted differences in height-for-age (regression coefficient), short-for-age and tall-for-age (log OR), compared to children at no risk of poverty. (1) Adjusted for gestational age, birth order, age of mother and region of mother’s birth.
(2) Adjusted for sex of child and age of child.
(3) Adjusted for sex of child, age of child, birth order, age of mother, region of mother’s birth and gestational age.
Adjusted effects for height-for-age based on linear growth curve models, for short-for-age and tall-for-age on logistic growth curve analysis.
(PDF)

S5 TableRandom effects linear regression growth curve results for mean height-for-age, and random effects logistic regression results for short-for-age and tall-for-age, including interaction terms between risk of poverty and age of child.Significance: *: *p* < 0.050, **: *p* < 0.010, ***: *p* < 0.001.For linear models: *σ*_*ν*:_: variance accounted for at child level; for logistic models: $\ln\left(\sigma_{\nu }^{2}\right)$: child level variance component, component value and, between parentheses, its standard error.*ρ*: proportion total variance due to child level variance component.(PDF)
