# Supplementary material for: Poverty and a child’s height development during early childhood: A double disadvantage? A study of the 2006–2009 birth cohorts in Flanders
Source: PLoS One. 2019 Jan 2;14(1):e0209170. doi: 10.1371/journal.pone.0209170 (PMC6314581; doi:10.1371/journal.pone.0209170)
Supplement: S1 Fig — (PDF) [file pone.0209170.s001.pdf]

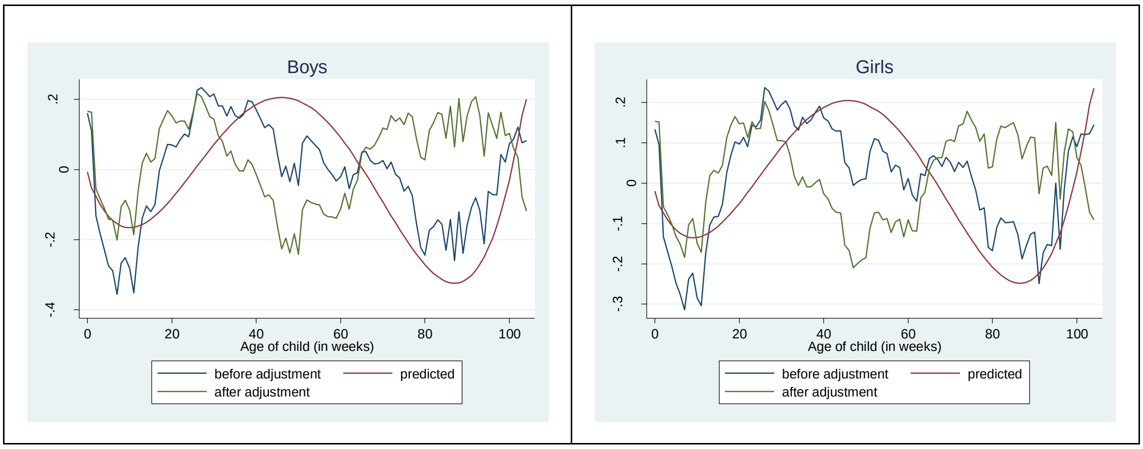

S1 Fig. Age and sex adjustment of standardized height-for-age scores: mean scores before adjustment, predicted means, and mean scores after adjustment, for boys and girls.
