# Supplementary material for: Poverty and a child’s height development during early childhood: A double disadvantage? A study of the 2006–2009 birth cohorts in Flanders
Source: PLoS One. 2019 Jan 2;14(1):e0209170. doi: 10.1371/journal.pone.0209170 (PMC6314581; doi:10.1371/journal.pone.0209170)
Supplement: S1 Table — Significance: *: p < .050, **: p < 0.010, ***: p < 0.0001. Models 1, 4a and 4b estimated using linear regression; models 2, 3, 5a, 5b, 6a and 6b estimated using logistic regression. (PDF) [file pone.0209170.s002.pdf]

S1 Table. Linear regression results for gestational age and length-at-birth, and logistic regression results for preterm birth, very preterm birth, short-at-birth, and very-short-at-birth.

	Gestational age	Preterm (< 37 wks)	Very preterm (< 32 wks)	Height-at-birth		Short-at-birth		Very-short-at-birth	
	(1)	(2)	(3)	(4a)	(4b)	(5a)	(5b)	(6a)	(6b)
Sex (1 = girl)	0.073*** (0.007)	-0.103*** (0.015)	-0.073 (0.041)	-0.790*** (0.010)	-0.855*** (0.008)	0.351*** (0.018)	0.716*** (0.024)	0.192*** (0.028)	0.579*** (0.041)
Birth order	-0.002 (0.004)	-0.141*** (0.009)	-0.143*** (0.024)	0.116*** (0.006)	0.118*** (0.004)	-0.172*** (0.011)	-0.119*** (0.013)	-0.256*** (0.018)	-0.167*** (0.023)
Age of mother	0.077*** (0.007)	-0.112*** (0.013)	-0.126*** (0.034)	0.171*** (0.010)	0.108*** (0.007)	-0.129*** (0.015)	-0.100*** (0.020)	-0.140*** (0.023)	-0.112** (0.033)
$(\text{Age of mother})^2$	-0.001*** (0.000)	0.002*** (0.000)	0.002*** (0.001)	-0.003*** (0.000)	-0.002*** (0.000)	0.003*** (0.000)	0.002*** (0.000)	0.003*** (0.000)	0.002*** (0.001)
Risk of poverty (ref = None (0))									
Low (1)	-0.117*** (0.014)	0.162*** (0.029)	-0.060 (0.085)	-0.379*** (0.020)	-0.262*** (0.015)	0.336*** (0.034)	0.326*** (0.042)	0.272*** (0.054)	0.309*** (0.075)
Medium (2)	-0.176*** (0.023)	0.263*** (0.046)	0.313** (0.117)	-0.574*** (0.032)	-0.408*** (0.025)	0.584*** (0.050)	0.617*** (0.063)	0.507*** (0.081)	0.544*** (0.116)
High (3+)	-0.261*** (0.017)	0.418*** (0.032)	0.488*** (0.081)	-0.741*** (0.023)	-0.493*** (0.018)	0.804*** (0.035)	0.757*** (0.045)	0.844*** (0.055)	0.819*** (0.078)

Region of mother's birth (ref = Belgium)									
Turkey	0.076** (0.022)	-0.151** (0.047)	0.064 (0.119)	-0.046 (0.031)	-0.114*** (0.024)	-0.163** (0.054)	-0.152* (0.070)	-0.064 (0.082)	-0.151 (0.121)
Morocco	0.419*** (0.018)	-0.408*** (0.041)	0.026 (0.097)	0.314*** (0.025)	-0.086*** (0.019)	-0.560*** (0.051)	-0.285*** (0.066)	-0.484*** (0.080)	-0.332** (0.121)
Western nations	0.188*** (0.021)	-0.141** (0.046)	-0.154 (0.128)	0.300*** (0.030)	0.142*** (0.023)	-0.209*** (0.057)	-0.136 (0.073)	-0.139 (0.086)	0.036 (0.121)
Southern Europe	0.182*** (0.026)	-0.265*** (0.057)	-0.233 (0.157)	-0.170*** (0.036)	-0.331*** (0.028)	-0.023 (0.060)	0.276*** (0.074)	-0.312** (0.107)	-0.127 (0.153)
Eastern Europe	0.177*** (0.024)	-0.099* (0.050)	0.078 (0.127)	0.281*** (0.034)	0.109*** (0.026)	-0.244*** (0.062)	-0.165* (0.079)	-0.205* (0.095)	-0.158 (0.141)
South, Latin and Central America	0.037 (0.045)	-0.320** (0.101)	-0.360 (0.291)	-0.072 (0.062)	-0.115* (0.048)	-0.432** (0.126)	-0.342* (0.158)	-0.566** (0.211)	-0.545 (0.304)
Asia + Oceania	0.062** (0.023)	-0.160** (0.049)	-0.364* (0.150)	-0.049 (0.032)	-0.097*** (0.025)	-0.159** (0.057)	0.003 (0.071)	-0.280** (0.094)	-0.048 (0.128)
Africa	0.088*** (0.023)	-0.059 (0.047)	0.208 (0.114)	0.008 (0.032)	-0.106*** (0.025)	-0.113* (0.055)	-0.030 (0.071)	-0.023 (0.083)	-0.002 (0.122)
Gestational age					0.910*** (0.002)		-0.927*** (0.006)		-0.987*** (0.009)
Constant	37.852*** (0.101)	-0.936*** (0.194)	-2.848*** (0.508)	47.493*** (0.141)	12.904*** (0.136)	-1.327*** (0.223)	33.031*** (0.365)	-2.115*** (0.341)	33.499*** (0.578)

N	267,251	267,251	267,251	263,891	263,773	263,891	263,773	263,891	263,773

Significance: *: $p < .050$, **: $p < 0.010$, ***: $p < 0.0001$.
Models 1, 4a and 4b estimated using linear regression; models 2, 3, 5a, 5b, 6a and 6b estimated using logistic regression.
