# Supplementary material for: Poverty and a child’s height development during early childhood: A double disadvantage? A study of the 2006–2009 birth cohorts in Flanders
Source: PLoS One. 2019 Jan 2;14(1):e0209170. doi: 10.1371/journal.pone.0209170 (PMC6314581; doi:10.1371/journal.pone.0209170)
Supplement: S2 Table — (1) Adjusted for sex of child, birth order, age of mother and region of mother’s birth. (2) Adjusted for sex of child, birth order, age of mother, region of mother’s birth and gestational age. Effects of risk of poverty are significant at p < 0.001 for all models. Adjusted means were calculated for gestational age and height-at-birth; adjusted proportions for preterm < 37 weeks, preterm < 32 weeks, short-at-birth < 46cm, very-short-at-birth < 44 cm. (PDF) [file pone.0209170.s003.pdf]

S2 Table. Observed and adjusted means and proportions for birth characteristics by risk of poverty.

		Risk of poverty			
		None (0)	Low (1)	Medium (2)	High (3+)
Mean gestational age	Observed	38.92 (38.91–38.93)	38.86 (38.84–38.88)	38.79 (38.75–38.84)	38.73 (38.70–38.76)
	Adjusted (1)	38.94 (38.93–38.94)	38.82 (38.79–38.85)	38.76 (38.72–38.80)	38.67 (38.64–38.71)
Preterm < 37 weeks	Observed	7.23% (7.12%–7.33%)	7.50% (7.15%–7.86%)	8.36% (7.72%–9.00%)	9.10% (8.67%–9.54%)
	Adjusted (1)	6.93% (6.82%–7.04%)	8.05% (7.66%–8.45%)	8.83% (8.12%–9.53%)	10.16% (9.62%–10.70%)
Preterm < 32 weeks	Observed	0.94% (0.90%–0.98%)	0.83% (0.71%–0.95%)	1.18% (0.93%–1.43%)	1.40% (1.22%–1.58%)
	Adjusted (1)	0.87% (0.83%–0.91%)	0.82% (0.69%–0.95%)	1.19% (0.92%–1.45%)	1.41% (1.20%–1.62%)
Height-at-birth	Observed	49.89 (49.88–49.90)	49.53 (49.49–49.56)	49.32 (49.26–49.39)	49.15 (49.11–49.19)
	Adjusted (1)	49.90 (49.88–49.91)	49.52 (49.48–49.55)	49.32 (49.26–49.38)	49.16 (49.11–49.20)
	Adjusted (2)	49.87 (49.86–49.88)	49.61 (49.58–49.63)	49.46 (49.41–49.51)	49.37 (49.34–49.41)

Short-at-birth < 46 cm	Observed	4.69% (4.60%–4.78%)	5.72% (5.41%–6.04%)	7.05% (6.46%–7.65%)	8.36% (7.93%–8.78%)
	Adjusted (1)	4.40% (4.31%–4.49%)	6.05% (5.70%–6.40%)	7.62% (6.95%–8.29%)	9.33% (8.79%–9.87%)
	Adjusted (2)	1.36% (1.31%–1.40%)	1.87% (1.72%–2.02%)	2.48% (2.19%–2.78%)	2.85% (2.61%–3.08%)
Very-short-at-birth < 44 cm	Observed	1.90% (1.84%–1.96%)	2.10% (1.90%–2.29%)	2.62% (2.24%–2.99%)	3.35% (3.08%–3.63%)
	Adjusted (1)	1.74% (1.68%–1.79%)	2.27% (2.05%–2.49%)	2.85% (2.43%–3.27%)	3.95% (3.58%–4.32%)
	Adjusted (2)	0.20% (0.18%–0.22%)	0.27% (0.23%–0.31%)	0.35% (0.27%–0.42%)	0.45% (0.39%–0.52%)

(1) Adjusted for sex of child, birth order, age of mother and region of mother's birth.
(2) Adjusted for sex of child, birth order, age of mother, region of mother's birth and gestational age.
Effects of risk of poverty are significant at $p < 0.001$ for all models.
Adjusted means were calculated for gestational age and length-at-birth; adjusted proportions for preterm < 37 weeks, preterm < 32 weeks, short-at-birth < 46cm, very-short-at-birth < 44 cm.
Figures between parentheses are 95% confidence intervals.
