# Supplementary material for: Poverty and a child’s height development during early childhood: A double disadvantage? A study of the 2006–2009 birth cohorts in Flanders
Source: PLoS One. 2019 Jan 2;14(1):e0209170. doi: 10.1371/journal.pone.0209170 (PMC6314581; doi:10.1371/journal.pone.0209170)
Supplement: S3 Table — Significance: *: p < 0.050, **: p < 0.010, ***: p < 0.001. Values in cells are estimated linear or logistic regression coefficients and, between parentheses, the standard errors of the coefficients. ln(σν2): child level variance component, component value and, between parentheses, its standard error. ρ: proportion total variance due to child level variance component. (PDF) [file pone.0209170.s004.pdf]

S3 Table. Random effects linear regression growth curve results for height-for-age, and random effects logistic regression growth curve results for short-for-age and tall-for-age.

b (s_b)	Height-for-age		Short-for-age			Tall-for-age		
	No controls	Controlled (1)	No controls	Controlled (2)	Controlled (3)	No controls	Controlled (2)	Controlled (3)
Risk of poverty (ref = 0, no risk)								
1, low risk	-0.068*** (0.007)	-0.081*** (0.007)	0.369*** (0.033)	0.412*** (0.034)	0.446*** (0.038)	-0.048 (0.035)	-0.027 (0.035)	-0.129** (0.037)
2, medium risk	-0.133*** (0.012)	-0.140*** (0.011)	0.737*** (0.052)	0.801*** (0.053)	0.808*** (0.059)	-0.047 (0.057)	-0.018 (0.057)	-0.157** (0.060)
3+, high risk	-0.180*** (0.008)	-0.176*** (0.008)	1.013*** (0.034)	1.107*** (0.034)	1.040*** (0.042)	0.039 (0.038)	0.081* (0.038)	-0.086* (0.043)
Sex (girl)				-0.398*** (0.019)	-0.391*** (0.020)		-0.252*** (0.018)	-0.303*** (0.019)
Age				-0.011*** (0.000)	-0.011*** (0.000)		-0.017*** (0.000)	-0.018*** (0.000)
$(Age)^2$				0.000*** (0.000)	0.000*** (0.000)		0.000*** (0.000)	0.000*** (0.000)
$(Age)^3$				0.000*** (0.000)	0.000*** (0.000)		0.000*** (0.000)	0.000*** (0.000)
$(Age)^4$				0.000*	0.000		0.000***	0.000***

				(0.000)	(0.000)		(0.000)	(0.000)
Gestational age		0.280*** (0.001)			-0.973*** (0.006)			0.642*** (0.008)
Age mother		0.024*** (0.003)			-0.142*** (0.018)			0.035 (0.018)
(Age mother)^2		0.000*** (0.000)			0.002*** (0.000)			0.000 (0.000)
Birth order		0.033*** (0.004)			-0.091*** (0.024)			0.009 (0.025)
(Birth order)^2		-0.006*** (0.001)			0.022*** (0.004)			-0.001 (0.004)
Region of mother’s birth								
Turkey		0.282*** (0.010)			-0.939*** (0.065)			0.835*** (0.052)
Morocco		0.089*** (0.008)			-0.327*** (0.051)			0.260*** (0.044)
Northern Europe + Western Europe + Northern America + Australia + New Zealand		0.095*** (0.010)			-0.225*** (0.064)			0.493*** (0.052)
Southern Europe		0.012 (0.012)			0.065 (0.070)			0.275*** (0.066)
Eastern Europe		0.277***			-0.865***			0.943***

		(0.012)			(0.076)			(0.055)
South, Latin and Central America + the Caribbean		0.058**<br>(0.021)			-0.225<br>(0.126)			0.126<br>(0.117)
Asia + Oceania		0.099***<br>(0.011)			-0.219**<br>(0.064)			0.429***<br>(0.058)
Africa		0.278***<br>(0.011)			-0.731***<br>(0.067)			0.997***<br>(0.053)
Constant	0.027***<br>(0.002)	-11.355***<br>(0.060)	-6.519***<br>(0.014)	-5.771***<br>(0.017)	34.481***<br>(0.350)	-5.812***<br>(0.020)	-5.072***<br>(0.022)	-31.279***<br>(0.419)
$\ln\left(\sigma_{\nu}^{2}\right)$			2.375<br>(0.006)	2.449<br>(0.006)	1.990<br>(0.012)	1.928<br>(0.010)	1.938<br>(0.010)	1.909<br>(0.011)
ρ	0.718	0.644	0.766<br>(0.001)	0.779<br>(0.001)	0.690<br>(0.003)	0.676<br>(0.002)	0.679<br>(0.002)	0.672<br>(0.002)

Significance: *: $p < 0.050$, **: $p < 0.010$, ***: $p < 0.001$.
Values in cells are estimated linear or logistic regression coefficients and, between parentheses, the standard errors of the coefficients.
$\ln\left(\sigma_{\nu}^{2}\right)$: child level variance component, component value and, between parentheses, its standard error.

ρ: proportion total variance due to child level variance component.
