# Supplementary material for: Poverty and a child’s height development during early childhood: A double disadvantage? A study of the 2006–2009 birth cohorts in Flanders
Source: PLoS One. 2019 Jan 2;14(1):e0209170. doi: 10.1371/journal.pone.0209170 (PMC6314581; doi:10.1371/journal.pone.0209170)
Supplement: S4 Table — (1) Adjusted for gestational age, birth order, age of mother and region of mother’s birth. (2) Adjusted for sex of child and age of child. (3) Adjusted for sex of child, age of child, birth order, age of mother, region of mother’s birth and gestational age. Adjusted effects for height-for-age based on linear growth curve models, for short-for-age and tall-for-age on logistic growth curve analysis. (PDF) [file pone.0209170.s005.pdf]

S4 Table. Observed and adjusted differences in height-for-age (regression coefficient), short-for-age and tall-for-age (log odds ratio), compared to children at no risk of poverty.

	Risk of poverty		
	Low	Medium	High
Height-for-age			
Observed	-0.068<br>(-0.083– -0.054)	-0.133<br>(-0.157– -0.109)	-0.180<br>(-0.196– -0.164)
Controlled (1)	-0.081<br>(-0.094– -0.068)	-0.140<br>(-0.161– -0.119)	-0.176<br>(-0.191– -0.160)
Short-for-age			
Observed	1.446<br>(1.355–1.544)	2.090<br>(1.888–2.314)	2.753<br>(2.577–2.942)
Controlled (2)	1.510<br>(1.414–1.614)	2.228<br>(2.010–2.471)	3.024<br>(2.827–3.235)
Controlled (3)	1.563<br>(1.450–1.684)	2.243<br>(1.999–2.516)	2.830<br>(2.607–3.071)
Tall-for-age			
Observed	0.953<br>(0.890–1.020)	0.954<br>(0.853–1.067)	1.040<br>(0.966–1.119)
Controlled (2)	0.973<br>(0.909–1.041)	0.982<br>(0.878–1.098)	1.084<br>(1.007–1.167)
Controlled (3)	0.879<br>(0.817–0.946)	0.854<br>(0.759–0.962)	0.918<br>(0.844–0.998)

(1) Adjusted for gestational age, birth order, age of mother and region of mother’s birth.
(2) Adjusted for sex of child and age of child.
(3) Adjusted for sex of child, age of child, birth order, age of mother, region of mother’s birth and gestational age.
Adjusted effects for height-for-age based on linear growth curve models, for short-for-age and tall-for-age on logistic growth curve analysis.
Figures between parentheses are 95% confidence intervals.
