# Supplementary material for: Poverty and a child’s height development during early childhood: A double disadvantage? A study of the 2006–2009 birth cohorts in Flanders
Source: PLoS One. 2019 Jan 2;14(1):e0209170. doi: 10.1371/journal.pone.0209170 (PMC6314581; doi:10.1371/journal.pone.0209170)
Supplement: S5 Table — Significance: *: p < 0.050, **: p < 0.010, ***: p < 0.001. For linear models: σν:: variance accounted for at child level; for logistic models: ln(σν2): child level variance component, component value and, between parentheses, its standard error. ρ: proportion total variance due to child level variance component. (PDF) [file pone.0209170.s006.pdf]

S5 Table. Random effects linear regression growth curve results for mean height-for-age, and random effects logistic regression results for short-for-age and tall-for-age, including interaction terms between risk of poverty and age of child.

	Mean height-for-age	Short-for-age	Tall-for-age
Sex (girl)		-0.414*** (0.027)	-0.515*** (0.025)
Age	-4.174E-04*** (3.070E-05)	-0.012*** (0.000)	-0.013*** (0.000)
$(Age)^2$	1.390E-06*** (2.190E-07)	2.670E-05*** (2.950E-06)	9.250E-05*** (3.270E-06)
$(Age)^3$	-2.670E-09*** (5.320E-10)	-2.430E-08** (7.400E-09)	-2.180E-07*** (8.040E-09)
$(Age)^4$	1.970E-12*** (3.980E-13)	7.240E-12 (5.670E-12)	1.570E-10*** (6.060E-12)
Risk of poverty (ref = 0, no risk)			
1, low risk	-0.214*** (0.008)	0.550*** (0.059)	-0.697*** (0.063)
2, medium risk	-0.312*** (0.012)	0.974*** (0.089)	-0.925*** (0.104)
3+, high risk	-0.381*** (0.009)	1.283*** (0.062)	-1.167*** (0.073)
Gestational age	0.280*** (0.001)	-1.202*** (0.009)	0.522*** (0.010)
Age mother	0.024*** (0.003)	-0.144*** (0.024)	-0.024 (0.023)
$(Age\ mother)^2$	-3.228E-04*** (5.480E-05)	0.003*** (0.000)	4.990E-04 (3.857E-04)
Birth order	0.032*** (0.004)	-0.312*** (0.030)	0.552*** (0.033)
$(Birth\ order)^2$	-0.006*** (0.001)	0.038*** (0.004)	-0.045*** (0.005)
Region of mother's birth			
Turkey	0.279*** (0.010)	-1.504*** (0.092)	1.319*** (0.059)
Morocco	0.084*** (0.008)	-0.937*** (0.073)	1.062*** (0.049)
Northern Europe + Western Europe + Northern America + Australia + New Zealand	0.095*** (0.010)	-0.059 (0.080)	0.481*** (0.069)
Southern Europe	0.009 (0.012)	-0.112 (0.092)	0.280** (0.086)
Eastern Europe	0.278*** (0.012)	-1.007*** (0.099)	0.922*** (0.071)
South, Latin and Central America + the Caribbean	0.059** (0.021)	-0.686*** (0.174)	0.524*** (0.142)
Asia + Oceania	0.098*** (0.011)	-0.180* (0.082)	0.511*** (0.074)
Africa	0.276*** (0.011)	-1.341*** (0.094)	1.589*** (0.059)
Interaction between risk of poverty and			
Age			

1, low risk	0.001***<br>(0.000)	-0.004**<br>(0.001)	0.014***<br>(0.001)
2, medium risk	0.001***<br>(0.000)	0.001<br>(0.002)	0.018***<br>(0.002)
3+, high risk	0.001***<br>(0.000)	0.002<br>(0.001)	0.022***<br>(0.001)
$(Age)^2$			
1, low risk	-1.150E-07<br>(6.970E-07)	3.130E-05**<br>(9.180E-06)	-6.360E-05***<br>(9.350E-06)
2, medium risk	1.440E-06<br>(1.160E-06)	6.770E-07<br>(1.300E-05)	-8.240E-05***<br>(1.440E-05)
3+, high risk	-1.020E-06<br>(7.550E-07)	2.160E-06<br>(7.920E-06)	-1.024E-04***<br>(9.630E-06)
$(Age)^3$			
1, low risk	-2.420E-09<br>(1.680E-09)	-7.090E-08**<br>(2.280E-08)	1.150E-07***<br>(2.250E-08)
2, medium risk	-6.830E-09*<br>(2.780E-09)	-1.350E-08<br>(3.230E-08)	1.600E-07***<br>(3.390E-08)
3+, high risk	1.030E-11<br>(1.800E-09)	-1.880E-08<br>(1.940E-08)	1.910E-07***<br>(2.270E-08)
$(Age)^4$			
1, low risk	1.720E-12<br>(1.260E-12)	5.090E-11**<br>(1.740E-11)	-7.120E-11***<br>(1.670E-11)
2, medium risk	5.080E-12*<br>(2.080E-12)	1.530E-11<br>(2.470E-11)	-1.060E-10***<br>(2.500E-11)
3+, high risk	-6.650E-13<br>(1.340E-12)	1.890E-11<br>(1.480E-11)	-1.210E-10***<br>(1.680E-11)
Constant	-11.327***<br>(0.060)	41.666***<br>(0.477)	-28.117***<br>(0.545)
$\sigma_\nu / \ln\left(\sigma_\nu^2\right)$	0.838***	2.331***<br>(0.018)	2.151***<br>(0.012)
ρ	0.645	0.758***<br>(0.003)	0.723***<br>(0.002)

Significance: *: $p < 0.050$, **: $p < 0.010$, ***: $p < 0.001$.

For linear models: σ_ν : variance accounted for at child level; for logistic models: $\ln\left(\sigma_\nu^2\right)$: child level variance component, component value and, between parentheses, its standard error.

ρ: proportion total variance due to child level variance component.
